# Disparities in Financial Strain for Older Adults and People With Disabilities in California

**DOI:** 10.1093/geroni/igab046.1372

**Published:** 2021-12-17

**Authors:** Lei Chen, Kathryn Kietzman, Rebecca Allen

**Affiliations:** 1 University of California, Los Angeles, Los Angeles, California, United States; 2 University of California, Los Angeles, UCLA Center for Health Policy Research, California, United States; 3 University of Alabama, Tuscaloosa, Alabama, United States

## Abstract

This study examines disparities in the experience of financial strain among older adults and people with disabilities by age, gender, race/ethnicity, poverty, and disability type. People with disabilities refer to those who report cognitive impairment, difficulties performing activities of daily living (ADLs) and/or instrumental activities of daily living (IADLs). Financial strain includes challenges that participants incurred during the last 12 months in acquiring food, housing, health care, or income. This study uses the data from the 2019 California Long-Term Services and Supports (LTSS) survey that was merged with data from the omnibus California Health Interview Survey (CHIS) (N=1097). This is the most comprehensive population-level dataset to examine LTSS needs, unmet needs, and uses of LTSS in California. Initial findings show that 50% of participants report spending less on food, while 40% report cutting down on saving for retirement, receiving and borrowing money from others, and experiencing a decline in household income. More than 20% note that they could not make rent or mortgage payments, had debt due to medical bills, and had to spend less on prescription medications or medical care. We also find significant disparities in financial strain by age, gender, poverty, and disability type; however, no significant disparities by race/ethnicity. This study is among the first to examine disparities in various financial strain types for people who need LTSS in California. The findings have policy implications for the Master Plan for Aging (MPA), which serves as a blueprint to build environments that promote an age-friendly California.

